# KDM4B Maintains Pyruvate Dehydrogenase Complex Expression to Support Mitochondrial Metabolism and Survival in Cancer Cells

**DOI:** 10.3390/epigenomes10030059

**Published:** 2026-09-16

**Authors:** Sachiko Sato, Arif Ul Hasan, Mami Obara, Eiichi Taira

**Affiliations:** Department of Pharmacology, School of Medicine, Iwate Medical University, Yahaba 028-3694, Japan

**Keywords:** KDM4B, pyruvate dehydrogenase complex, cancer metabolism, epigenetic regulation, histone demethylase, mitochondrial metabolism, metabolic reprogramming

## Abstract

**Background/Objectives:** Metabolic reprogramming is a hallmark of cancer and requires coordinated regulation of glycolysis and mitochondrial metabolism. The pyruvate dehydrogenase complex (PDC) links these pathways by catalyzing the conversion of pyruvate to acetyl-CoA, yet the epigenetic mechanisms regulating PDC expression remain poorly understood. We investigated whether the histone demethylase KDM4B regulates PDC expression and mitochondrial metabolism in cancer cells. **Methods:** Human colorectal carcinoma (HCT-116), cervical adenocarcinoma (HeLa), melanoma (G-361), and non-malignant human proximal tubule epithelial (HK-2) cells were analyzed using glucose-response assays, RNA sequencing, RT-qPCR, immunoblotting, metabolic assays, fluorescence imaging, and cell viability analyses. KDM4B was disrupted by small interfering RNA (siRNA) and the selective inhibitor NCGC00244536. **Results:** Glucose availability induced coordinated upregulation of the PDC subunits PDHA1, DLD, and DLAT and was associated with increased proliferation and KDM4B expression in cancer cells. Both genetic depletion and pharmacological inhibition of KDM4B suppressed PDC expression and increased the repressive histone mark H3K9me3. Exploratory RNA-seq analysis revealed coordinated metabolic transcriptional reprogramming characterized by induction of glycolytic genes and suppression of PDC, the tricarboxylic acid cycle, and electron transport chain genes, together with increased PDK1 and reduced PDP1 expression, consistent with impaired mitochondrial glucose oxidation. These changes were accompanied by reduced pyruvate dehydrogenase activity, extracellular pyruvate accumulation, ATP depletion, impaired glucose uptake, mitochondrial depolarization, caspase-3 activation, increased lactate dehydrogenase release, reduced viability, and diminished proliferative capacity. These effects were generally more pronounced in the cancer cell models than in HK-2 cells. **Conclusions:** Our findings identify KDM4B as a regulator associated with maintenance of PDC expression and mitochondrial glucose metabolism in cancer cells. KDM4B inhibition suppresses PDC abundance and activity and is accompanied by metabolic dysfunction, mitochondrial depolarization, and reduced cell survival. These findings support a model in which the KDM4B–PDC axis may contribute to metabolic homeostasis and survival in cancer cells and suggest that this pathway may represent a potential therapeutic vulnerability.

## 1. Introduction

Metabolic reprogramming is a fundamental hallmark of cancer that enables malignant cells to sustain proliferation, adapt to microenvironmental stress, and survive under conditions of fluctuating nutrient availability [1,2,3]. Rather than relying exclusively on mitochondrial oxidative phosphorylation for ATP production, many cancer cells reprogram glucose metabolism to support ATP generation as well as the biosynthetic demands of rapid proliferation. This metabolic phenotype, historically described as the Warburg effect, is now recognized as considerably more complex than originally proposed. Accumulating evidence indicates that many solid tumors retain functional mitochondrial metabolism and dynamically coordinate glycolysis with oxidative phosphorylation to maximize metabolic flexibility and support tumor progression [3,4,5].

At the center of this metabolic network is the pyruvate dehydrogenase complex (PDC), which catalyzes the irreversible conversion of pyruvate into acetyl-CoA, thereby linking cytosolic glycolysis with the mitochondrial tricarboxylic acid (TCA) cycle. The PDC is composed of three catalytic components: pyruvate dehydrogenase (E1; PDHA1), dihydrolipoamide acetyltransferase (E2; DLAT), and dihydrolipoamide dehydrogenase (E3; DLD). As the principal gateway linking glycolysis to mitochondrial oxidative metabolism, the PDC has emerged as a critical regulator of cancer metabolism. Previous studies have primarily focused on the post-translational regulation of PDH activity through the opposing actions of pyruvate dehydrogenase kinases (PDKs), which inhibit the complex, and pyruvate dehydrogenase phosphatases (PDPs), which activate it [6,7,8,9]. In contrast, considerably less is known about the transcriptional mechanisms regulating the expression of the core structural components of the PDC.

Increasing evidence suggests that metabolic remodeling in cancer is closely integrated with epigenetic regulation. Among the enzymes coordinating these processes, the Jumonji C (JmjC) domain-containing histone lysine demethylase KDM4B (JMJD2B) has emerged as an important regulator of tumor progression, metabolic adaptation, stemness, and therapeutic resistance across multiple malignancies [10,11,12,13]. KDM4B primarily demethylates the transcriptionally repressive histone H3 lysine 9 trimethylation (H3K9me3) mark, thereby facilitating transcriptional activation of target genes [12].

In colorectal cancer, KDM4B has been shown to stimulate AKT activation via TRAF6-mediated ubiquitination, which promotes glucose metabolism and upregulates the transporter GLUT1 [14]. Furthermore, KDM4B acts as a critical coactivator for c-Myc, maintaining the Warburg phenotype, while its inhibition forces a metabolic switch toward OXPHOS [15]. Beyond direct nuclear activation, recent research has identified a mitochondrial-to-nuclear retrograde signaling axis in colorectal cancer (the [Ca^2+^]m-PDP1-PDH axis), where alterations in mitochondrial PDH activity directly influence the nuclear epigenetic landscape [16]. However, despite these important observations, whether KDM4B regulates the transcriptional maintenance of the core structural components of the pyruvate dehydrogenase complex—and thereby coordinates mitochondrial glucose oxidation—has not been investigated.

In the present study, we examined the relationship between KDM4B and the PDC using complementary genetic and pharmacological approaches in multiple human cancer cell models together with a non-malignant epithelial cell line. Our findings identify KDM4B as a previously unrecognized epigenetic regulator associated with PDC expression and mitochondrial glucose metabolism in cancer cells. We propose that KDM4B contributes to the coordination of glycolytic glucose utilization and mitochondrial oxidative metabolism, such that its inhibition disrupts metabolic homeostasis and more strongly affects the survival of the cancer cell models examined than that of the non-malignant cell line.

## 2. Results

### 2.1. Glucose Availability Promotes Proliferation and Induces Pyruvate Dehydrogenase Complex Gene Expression

**Figure 1:**
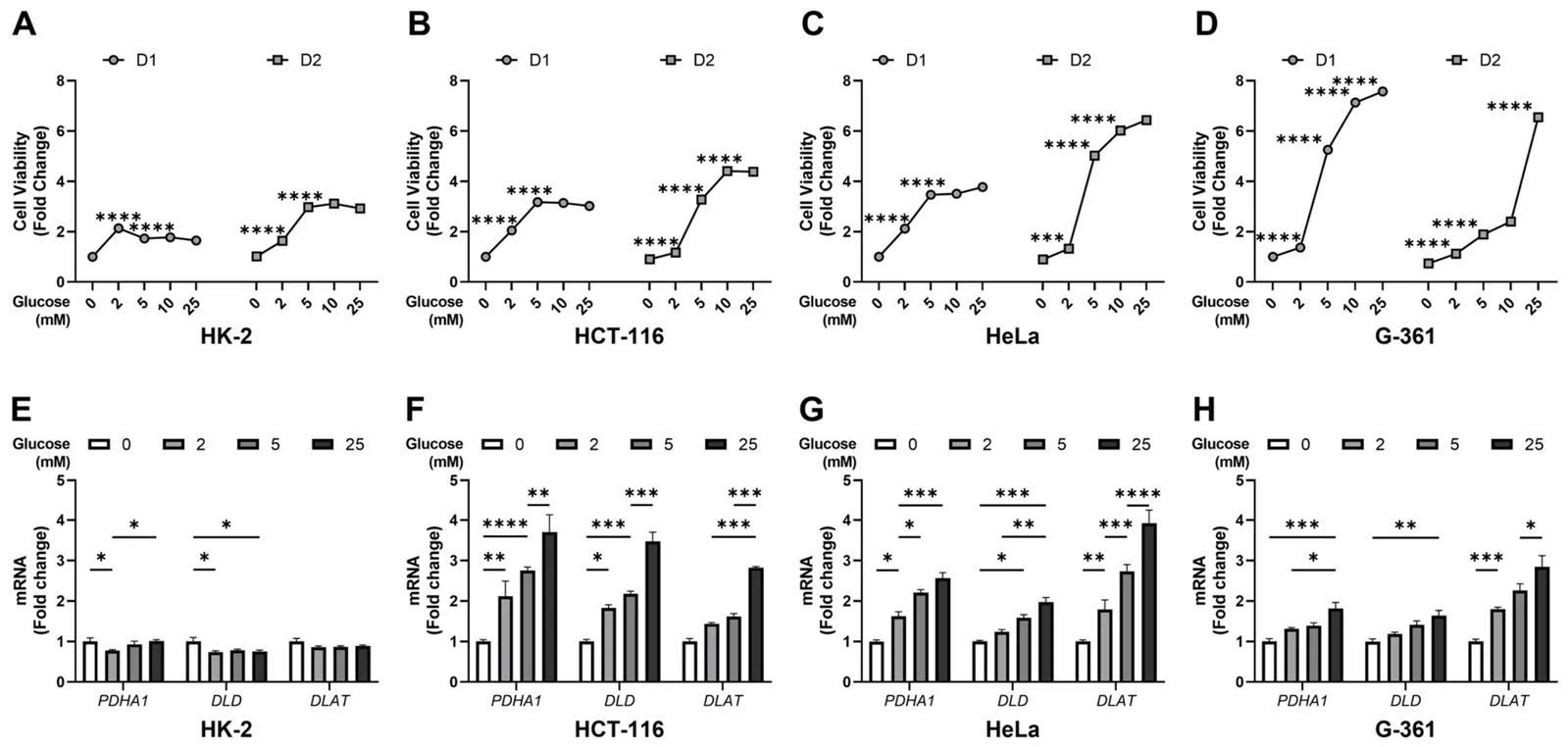
Glucose dose-dependently enhances cell viability and upregulates expression of genes encoding the pyruvate dehydrogenase complex. Human non-malignant proximal tubule epithelial cells (HK-2) and human cancer cell lines (HCT-116, HeLa, and G-361) were cultured in glucose-free medium (0 mM glucose) or treated with increasing concentrations of glucose (2, 5, 10, and 25 mM). (**A**–**D**) Cell viability was assessed using the WST-8 assay at 24 h (D1) and 48 h (D2) following treatment (*N* = 4). (**E**–**H**) Cells were exposed to the indicated glucose concentrations for 16 h before RNA extraction. Relative mRNA levels of *PDHA1*, *DLD*, and *DLAT* were determined by RT-qPCR (*N* = 3). Data are presented as mean ± SEM and expressed as fold change relative to the corresponding 0 mM glucose group (set to 1.0). Statistical significance was determined using two-way ANOVA followed by Tukey’s multiple-comparisons test for panels (**A**–**D)** and one-way ANOVA followed by Tukey’s multiple-comparisons test for panels (**E**–**H**). * *p* < 0.05; ** *p* < 0.01; *** *p* < 0.001; **** *p* < 0.0001 compared to the respective 0 mM glucose group. *N* denotes the number of independent biological replicates from one representative experiment.

To establish a glucose-dependent metabolic model, we first examined the effects of glucose availability on cell proliferation in non-malignant and cancer cell lines. Increasing glucose concentrations promoted a robust, dose-dependent increase in cell viability in all three cancer cell lines (HCT-116, HeLa, and G-361) at both 24 h (D1) and 48 h (D2) (Figure 1B–D). Although glucose supplementation also increased the viability of non-malignant HK-2 cells (Figure 1A), the proliferative response was considerably less pronounced than that observed in the cancer cell lines. At 48 h (D2), cell viability increased by approximately 4- to 8-fold in the cancer cell lines at 25 mM glucose compared with the glucose-free control, whereas HK-2 cells exhibited an increase of approximately 3-fold.

To investigate whether this enhanced proliferative response was accompanied by alterations in mitochondrial glucose metabolism, we next examined the expression of genes encoding the core structural components of the pyruvate dehydrogenase complex (PDC). RT-qPCR analysis demonstrated a robust, dose-dependent increase in the expression of *PDHA1*, *DLD*, and *DLAT* in all three cancer cell lines following glucose supplementation (Figure 1F–H). In contrast, HK-2 cells exhibited little or no transcriptional induction in response to increasing glucose concentrations. Instead, the expression of *PDHA1* and *DLD* showed a slight downward trend, whereas the expression of *DLAT* remained largely unchanged (Figure 1E).

Taken together, these findings demonstrate that glucose availability was associated with greater increases in both cell proliferation and the expression of genes encoding the core components of the PDC in the cancer cell models examined than in HK-2 cells, which exhibited comparatively limited transcriptional and proliferative responses under these conditions.

### 2.2. Glucose Availability Induces KDM4B Expression and Modulates PDH Phosphorylation Signals in Cancer Cells

Because glucose availability increased PDC gene expression (Figure 1), we next investigated whether it also regulates the expression of the histone demethylase KDM4B and the protein status of the core PDC subunit PDH E1α (hereafter PDH). Cells were cultured under glucose-free (0 mM) or high-glucose (25 mM) conditions, and KDM4B expression and total and phosphorylated PDH levels were evaluated.

**Figure 2:**
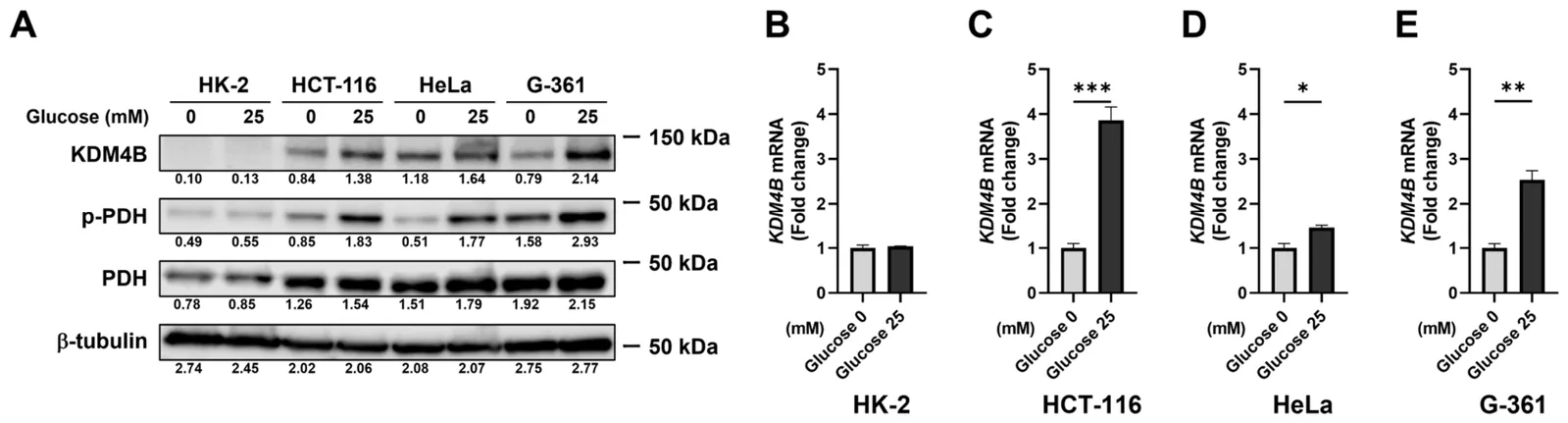
Glucose availability selectively upregulates KDM4B expression and increases PDH phosphorylation in cancer cell lines. Human non-malignant proximal tubule epithelial cells (HK-2) and human cancer cell lines (HCT-116, HeLa, and G-361) were cultured in glucose-free medium (0 mM glucose) or high-glucose medium (25 mM glucose) for 16 h. (**A**) Representative immunoblots of whole-cell lysates showing KDM4B, phospho-PDH (p-PDH Ser293), and total PDH, with β-tubulin as the loading control. Numbers below the bands indicate densitometric intensity values obtained from the blot. (**B**–**E**) Relative KDM4B mRNA expression levels were determined by RT-qPCR in (**B**) HK-2, (**C**) HCT-116, (**D**) HeLa, and (**E**) G-361 cells (*N* = 3). Data are presented as mean ± SEM and expressed as fold change relative to the corresponding 0 mM glucose group (set to 1.0). Statistical significance was determined using an unpaired two-tailed Student’s *t*-test. * *p* < 0.05; ** *p* < 0.01; *** *p* < 0.001 compared with the corresponding 0 mM glucose group. *N* denotes the number of independent biological replicates from one representative experiment.

The representative immunoblot showed that KDM4B protein expression was barely detectable in non-malignant HK-2 cells and remained largely unchanged following glucose supplementation (Figure 2A). In contrast, all three cancer cell lines (HCT-116, HeLa, and G-361) exhibited detectable basal KDM4B expression, which increased under high-glucose conditions. This increase was particularly apparent in G-361 melanoma cells (Figure 2A). Consistent with the protein data, RT-qPCR analysis demonstrated that glucose significantly increased KDM4B mRNA expression in HCT-116, HeLa, and G-361 cells, whereas KDM4B transcript levels remained unchanged in HK-2 cells (Figure 2B–E). Among the cancer cell lines, the greatest induction was observed in HCT-116 and G-361 cells (Figure 2C,E).

We next examined whether these changes were accompanied by alterations in PDH protein abundance and phospho-PDH (Ser293) signals. In HK-2 cells, the representative immunoblot showed little change in total PDH or p-PDH (Ser293) following glucose supplementation (Figure 2A). In contrast, high-glucose conditions were associated with a modest increase in total PDH protein abundance in HCT-116, HeLa, and G-361 cells, consistent with the glucose-dependent upregulation of PDC-related genes observed in Figure 1. The immunoblot also showed increased phospho-PDH (Ser293) signals under high-glucose conditions in the three cancer cell lines (Figure 2A).

Overall, these findings suggest that glucose availability is associated with increased KDM4B expression in the cancer cell models examined and is accompanied by changes in PDH protein abundance and phospho-PDH (Ser293) signals, whereas non-malignant HK-2 cells exhibited comparatively limited responses.

### 2.3. Genetic Depletion Identifies KDM4B as an Upstream Regulator of Pyruvate Dehydrogenase Complex in Cancer Cells

**Figure 3:**
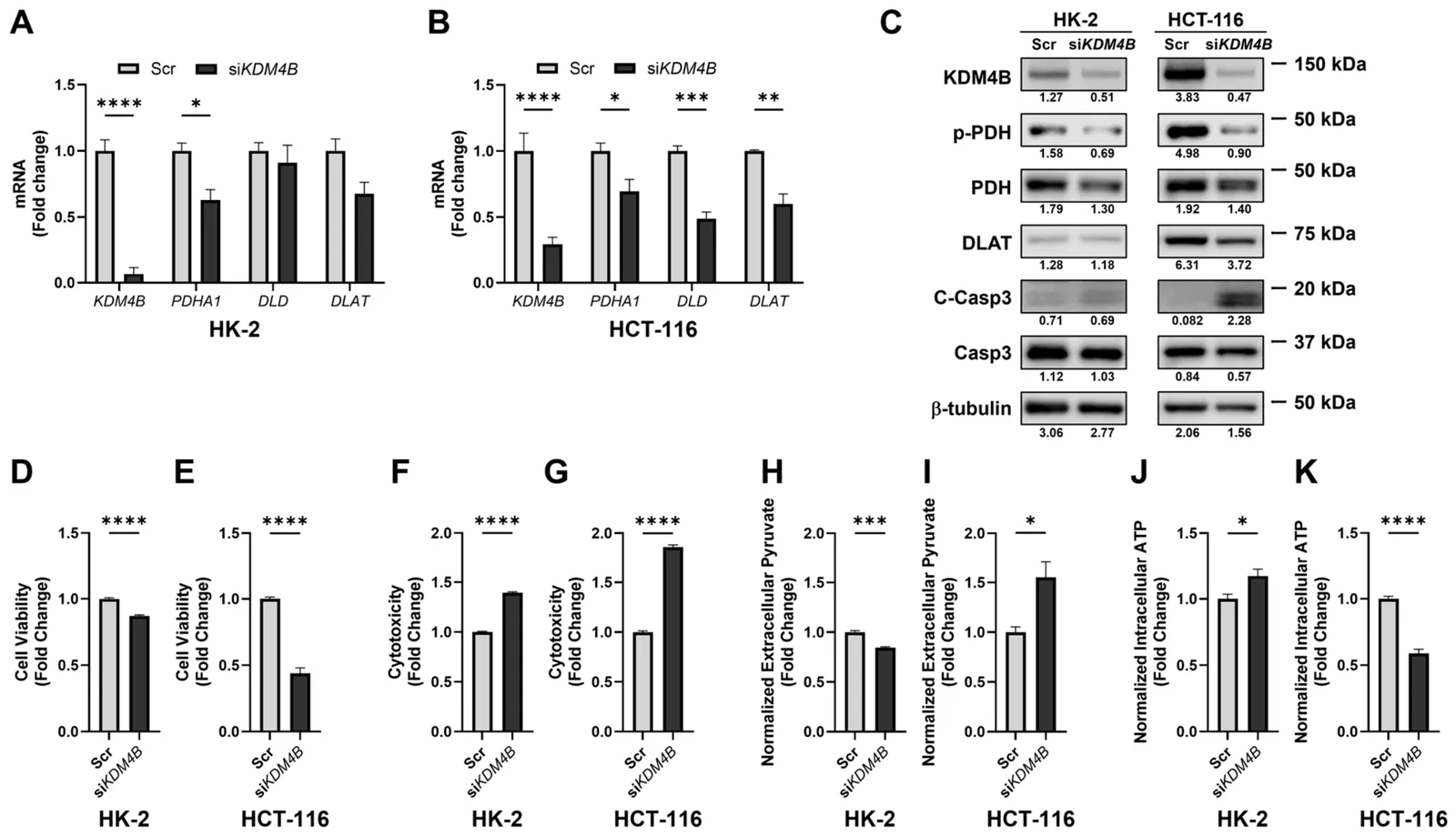
Genetic depletion of KDM4B suppresses pyruvate dehydrogenase complex expression and selectively disrupts cancer cell metabolic homeostasis. Human non-malignant proximal tubule epithelial cells (HK-2) and colorectal carcinoma cells (HCT-116) were transfected with scrambled negative control siRNA (Scr) or KDM4B-specific siRNA (si*KDM4B*). (**A**,**B**) Relative mRNA levels of *KDM4B* and the pyruvate dehydrogenase complex (PDC) core subunits *PDHA1*, *DLD*, and *DLAT* were determined by RT-qPCR four days post-transfection (*N* = 3). (**C**) Representative immunoblots of whole-cell lysates showing KDM4B, phospho-PDH (p-PDH Ser293), total PDH, DLAT, cleaved caspase-3 (C-Casp3), and total caspase-3 (Casp3), with β-tubulin as the loading control. Numbers below the bands indicate densitometric intensity values obtained from the blot. (**D**,**E**) Cell viability was assessed using the CellTiter-Glo Cell Viability Assay (*N* = 4). (**F**,**G**) Cellular cytotoxicity was evaluated by measuring lactate dehydrogenase (LDH) release into the culture supernatant (*N* = 4). (**H**,**I**) Extracellular pyruvate and (**J**,**K**) intracellular ATP levels were quantified and normalized to viable cell number determined in parallel cultures (*N* = 4). Data are presented as mean ± SEM and expressed as fold change relative to the corresponding Scr group (set to 1.0). Statistical significance was determined using an unpaired two-tailed Student’s *t*-test. * *p* < 0.05; ** *p* < 0.01; *** *p* < 0.001; **** *p* < 0.0001 compared with the corresponding Scr group. *N* denotes the number of independent biological replicates from one representative experiment.

Given the established role of KDM4B in tumor progression and metabolic adaptation, we next investigated whether KDM4B is associated with PDC expression and metabolic function. To this end, KDM4B was selectively depleted using small interfering RNA (si*KDM4B*) in non-malignant HK-2 cells and HCT-116 colorectal cancer cells. RT-qPCR and immunoblot analyses demonstrated efficient KDM4B depletion at both the mRNA and protein levels in both cell lines (Figure 3A–C).

KDM4B depletion differentially affected the expression of PDC core subunits in non-malignant and malignant cells. In HCT-116 cells, silencing *KDM4B* significantly reduced the expression of all three core PDC structural subunits, *PDHA1*, *DLD*, and *DLAT* (Figure 3B). In contrast, HK-2 cells exhibited a significant reduction in *PDHA1* expression, whereas *DLD* and *DLAT* expression showed only modest, non-significant changes (Figure 3A). The representative immunoblot showed reduced KDM4B, total PDH, and DLAT protein abundance following si*KDM4B* treatment (Figure 3C). Phospho-PDH (Ser293) was also reduced following KDM4B depletion. Furthermore, the immunoblot showed increased cleaved caspase-3 in HCT-116 cells following KDM4B depletion, whereas cleaved caspase-3 was not detectable in HK-2 cells (Figure 3C).

To determine whether these molecular alterations were associated with functional changes in cell survival, we next assessed cell viability and membrane damage. KDM4B depletion significantly reduced the viability of HCT-116 cells by more than 50%, whereas only a modest reduction was observed in HK-2 cells (Figure 3D,E). Consistent with these findings, lactate dehydrogenase (LDH) release was markedly increased in HCT-116 cells but showed only a slight increase in HK-2 cells (Figure 3F,G).

Because KDM4B depletion altered PDC expression, we next examined the associated metabolic changes. In HCT-116 cells, KDM4B depletion significantly increased extracellular pyruvate levels and markedly reduced intracellular ATP levels (Figure 3I,K). In contrast, HK-2 cells exhibited a distinct metabolic response characterized by reduced extracellular pyruvate levels together with a modest but significant increase in intracellular ATP levels (Figure 3H,J).

Overall, these findings identify KDM4B as an upstream regulator associated with PDC expression and metabolic homeostasis in HCT-116 cells. Genetic depletion of KDM4B selectively disrupted pyruvate metabolism, reduced ATP production, and promoted apoptotic cell death in cancer cells while exerting comparatively modest—and metabolically distinct—effects in non-malignant HK-2 cells.

### 2.4. KDM4B Inhibition Induces Coordinated Metabolic Transcriptional Reprogramming

To gain mechanistic insight into the transcriptional changes underlying the alterations in PDH activity and ATP production following KDM4B inhibition, we performed RNA sequencing (RNA-seq) in HCT-116 cells cultured under glucose-free conditions, 25 mM glucose, or 25 mM glucose supplemented with 5 μM NCGC00244536 (a selective KDM4B inhibitor) [13].

**Figure 4:**
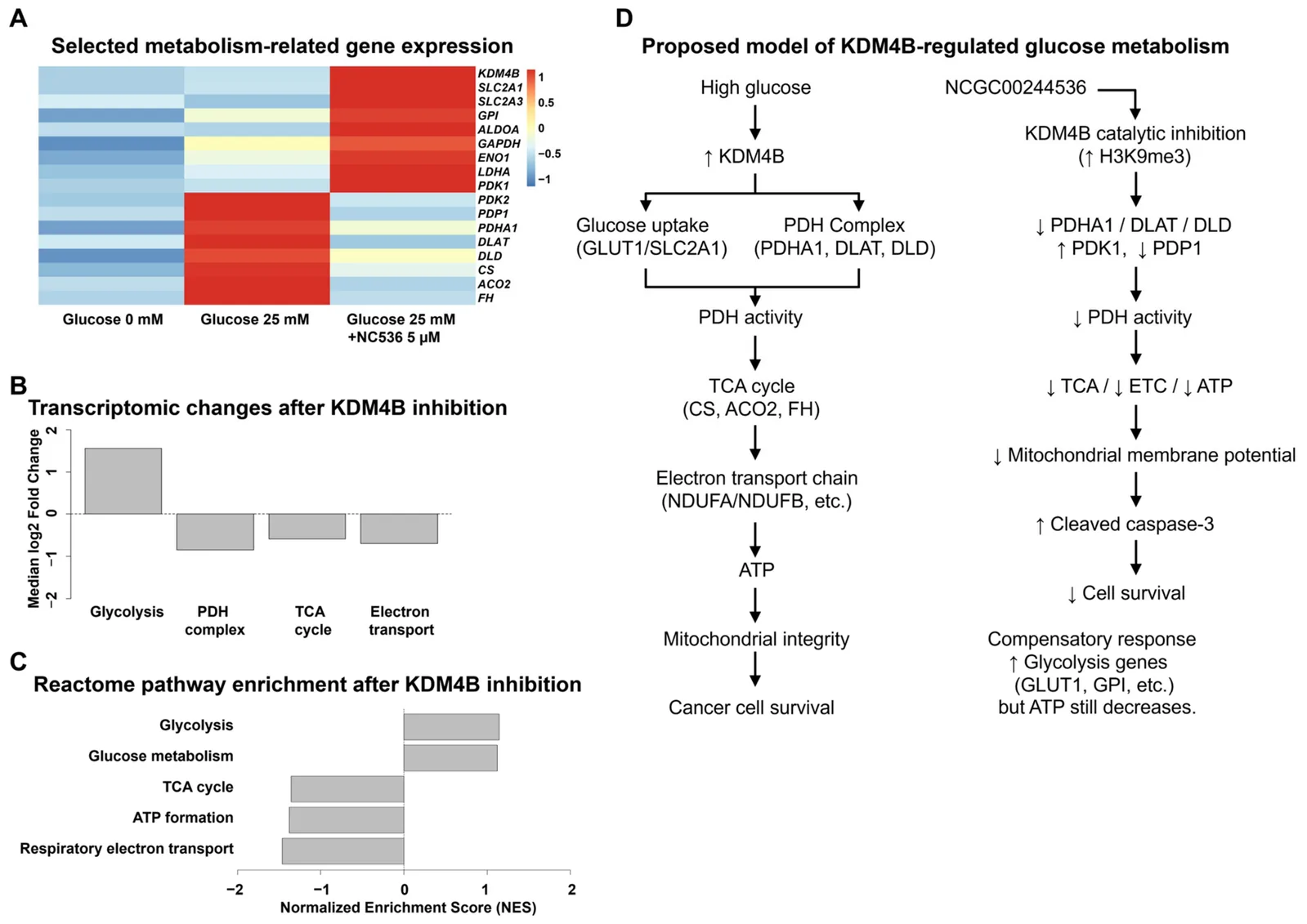
Transcriptomic analysis reveals coordinated metabolic reprogramming following KDM4B inhibition. (**A**) Heatmap showing the expression of representative genes involved in glucose uptake, glycolysis, pyruvate dehydrogenase (PDH) regulation, the PDH complex, and the tricarboxylic acid (TCA) cycle in HCT-116 cells cultured under glucose-free medium (0 mM), 25 mM glucose, and 25 mM glucose plus 5 μM NCGC00244536 (NC536) conditions. (**B**) Summary of pathway-specific transcriptional changes represented as the median log2 fold change following KDM4B inhibition relative to the glucose-treated group. (**C**) Gene Set Enrichment Analysis (GSEA) using Reactome pathways demonstrates a trend toward suppression of mitochondrial oxidative metabolism, including respiratory electron transport, ATP formation, and the TCA cycle, accompanied by relative enrichment of glycolytic pathways. (**D**) Proposed working model illustrating the coordinated metabolic consequences of KDM4B inhibition. Pharmacological inhibition of KDM4B suppresses expression of the pyruvate dehydrogenase complex (PDC) subunits PDHA1, DLAT, and DLD while increasing PDK1 expression, resulting in reduced PDH activity, impaired mitochondrial oxidative metabolism, decreased ATP production, and activation of compensatory glycolytic gene expression. Arrows indicate increases/upregulation (↑) or decreases/downregulation (↓) in the indicated molecular or metabolic processes.

A focused analysis of glucose metabolism revealed coordinated transcriptional alterations across multiple steps of cellular energy metabolism (Figure 4A,B, and Supplementary Table S1). Several glycolytic genes, including *GPI*, *GAPDH*, *ENO1*, *ALDOA*, and *LDHA*, were upregulated following KDM4B inhibition, suggesting enhanced glycolytic capacity. In contrast, expression of multiple components of the pyruvate dehydrogenase complex, including *PDHA1*, *DLAT*, and *DLD*, was reduced. In parallel, the PDH kinase *PDK1* was markedly upregulated, whereas the PDH phosphatase *PDP1* was downregulated, indicating transcriptional changes predicted to suppress PDH activity (Figure 4A,B, Supplementary Table S2).

Beyond the PDH complex, several genes associated with the tricarboxylic acid (TCA) cycle also exhibited reduced expression (Figure 4B, and Supplementary Table S3). Likewise, genes encoding components of the mitochondrial electron transport chain showed an overall decrease (median log2 fold change ≈ −0.70), suggesting a coordinated reduction in mitochondrial oxidative metabolism (Figure 4A,B, Supplementary Table S4).

To further evaluate pathway-level alterations, we performed Reactome gene set enrichment analysis (GSEA). Pathways associated with mitochondrial oxidative metabolism, including respiratory electron transport, ATP formation by chemiosmotic coupling, and the TCA cycle, exhibited negative enrichment trends, whereas glycolysis and glucose metabolism showed positive enrichment trends (Figure 4C, Supplementary Table S5). Although these enrichments did not reach statistical significance, likely because this exploratory RNA-seq analysis was performed without biological replicates, the direction of pathway changes closely paralleled the biochemical alterations observed in this study, including altered pyruvate metabolism and reduced ATP production. Together, these transcriptomic findings are consistent with a coordinated metabolic shift away from mitochondrial glucose oxidation toward glycolytic metabolism following KDM4B inhibition.

### 2.5. Pharmacological Inhibition of KDM4B Suppresses Pyruvate Dehydrogenase Complex Expression and Restores Repressive Histone Methylation

**Figure 5:**
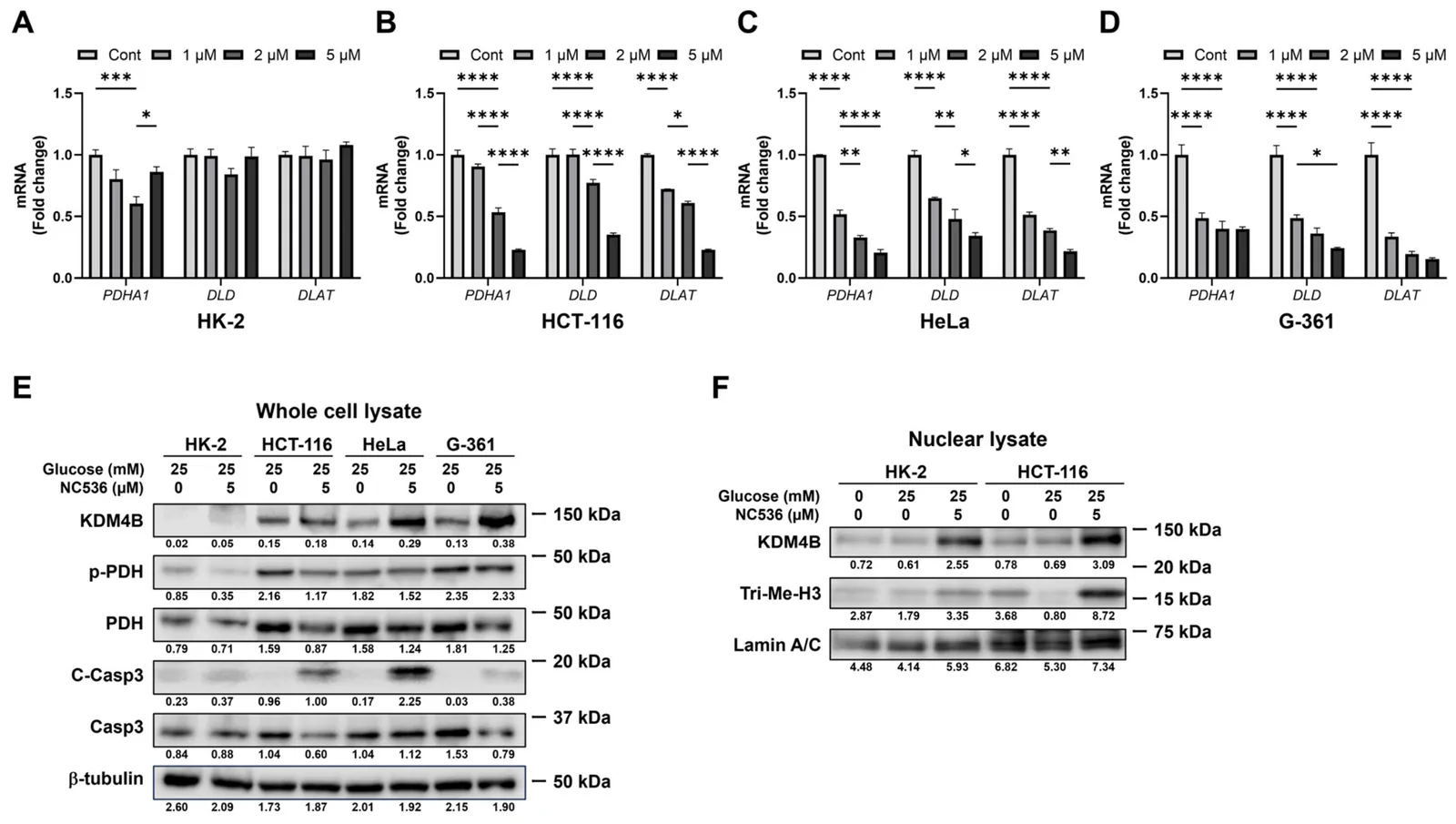
Pharmacological inhibition of KDM4B suppresses pyruvate dehydrogenase complex expression and restores repressive histone methylation in cancer cells. Human non-malignant proximal tubule epithelial cells (HK-2) and human cancer cell lines (HCT-116, HeLa, and G-361) were cultured in high-glucose medium (25 mM glucose) and treated with vehicle (DMSO; Cont) or increasing concentrations of NCGC00244536 (1, 2, and 5 µM) for 16 h. (**A**–**D**) Relative mRNA levels of the pyruvate dehydrogenase complex (PDC) core subunits PDHA1, DLD, and DLAT were determined by RT-qPCR (*N* = 3). Data are presented as mean ± SEM and expressed as fold change relative to the corresponding vehicle control group (set to 1.0). Statistical significance was determined using one-way ANOVA followed by Tukey’s multiple-comparisons test. * *p* < 0.05; ** *p* < 0.01; *** *p* < 0.001; **** *p* < 0.0001 compared with the corresponding vehicle control group. *N* denotes the number of independent biological replicates from one representative experiment. (**E**) Representative immunoblots of whole-cell lysates from HK-2, HCT-116, HeLa, and G-361 cells cultured in high-glucose medium (25 mM glucose) and treated with vehicle (DMSO; 0 µM) or 5 µM NCGC00244536 (NC536) for 16 h, showing KDM4B, phospho-PDH (p-PDH Ser293), total PDH, cleaved caspase-3 (C-Casp3), and total caspase-3 (Casp3), with β-tubulin as the loading control. (**F**) Representative immunoblots of nuclear lysates from HK-2 and HCT-116 cells cultured under glucose-free (0 mM glucose) or high-glucose (25 mM glucose) conditions in the absence or presence of 5 µM NCGC00244536 for 16 h, showing KDM4B, trimethylated histone H3 (Tri-Me-H3) and Lamin A/C as the nuclear loading control. Numbers below the bands indicate densitometric intensity values obtained from the blots.

To examine whether the transcriptomic findings were reflected at the protein level and to further investigate the molecular effects of pharmacological KDM4B inhibition, we next examined its impact on PDC expression and histone methylation status (Figure 5). NCGC00244536 treatment induced a dose-dependent reduction in the mRNA expression of the core PDC subunits *PDHA1*, *DLD*, and *DLAT* in all three cancer cell lines (HCT-116, HeLa, and G-361) (Figure 5B–D). In contrast, HK-2 cells exhibited only a modest reduction in *PDHA1* expression, whereas *DLD* and *DLAT* expression remained largely unchanged following treatment (Figure 5A). These findings closely resembled the transcriptional effects observed following genetic depletion of KDM4B.

We next examined whether these transcriptional alterations were reflected at the protein level. HK-2, HCT-116, HeLa, and G-361 cells were cultured under high-glucose (25 mM) conditions in the absence or presence of 5 μM NCGC00244536 (Figure 5E,F). The representative immunoblots showed increased KDM4B protein abundance following NCGC00244536 treatment in HCT-116, HeLa, and G-361 cells, with a smaller change in HCT-116 cells (Figure 5E). Total PDH protein abundance was reduced in the three cancer cell lines, consistent with the transcriptional suppression of PDC components observed by RT-qPCR and RNA sequencing. Phospho-PDH (Ser293) levels also decreased following NCGC00244536 treatment, although this reduction was less pronounced in HeLa and G-361 cells than in HCT-116 cells. In contrast, total PDH protein levels in non-malignant HK-2 cells were minimally affected by inhibitor treatment. NCGC00244536 treatment was also associated with increased cleaved caspase-3 signals in the cancer cell lines, particularly in HCT-116 and HeLa cells, whereas the change appeared less pronounced in G-361 cells (Figure 5E).

Because KDM4B functions as a histone demethylase, we next assessed whether pharmacological inhibition altered histone methylation status. A similar increase in KDM4B protein was also detected in the nuclear fraction of HCT-116 cells following NCGC00244536 treatment (Figure 5F). Under high-glucose conditions, trimethylated histone H3 levels were reduced compared with glucose-free conditions in HCT-116 cells, whereas NCGC00244536 treatment restored the levels (Figure 5F). By contrast, trimethylated histone H3 levels in HK-2 cells showed little change in response to either glucose availability or NCGC00244536 treatment.

Taken together, these findings demonstrate that pharmacological inhibition of KDM4B recapitulates the effects of genetic depletion by suppressing PDC expression at both the transcriptional and protein levels while restoring repressive histone methylation in cancer cells. These molecular alterations were accompanied by activation of apoptotic signaling, supporting the selective cytotoxic effects of KDM4B inhibition.

### 2.6. Pharmacological Inhibition of KDM4B Impairs PDH Activity and Disrupts Cellular Energy Metabolism

**Figure 6:**
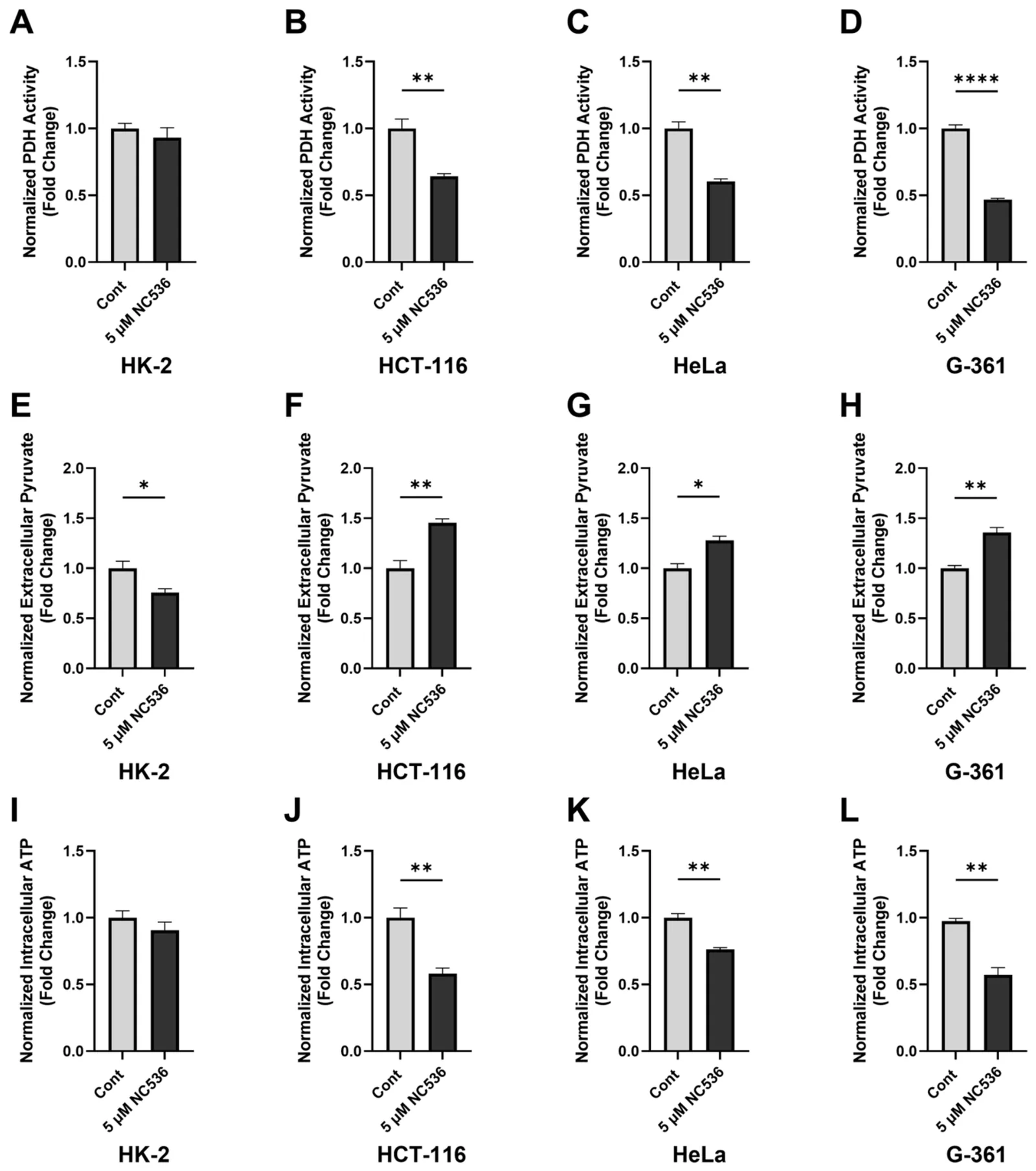
Pharmacological inhibition of KDM4B selectively disrupts pyruvate dehydrogenase complex activity, promotes extracellular pyruvate accumulation, and depletes intracellular ATP in cancer cells. Human non-malignant proximal tubule epithelial cells (HK-2) and human cancer cell lines (HCT-116, HeLa, and G-361) were cultured in high-glucose medium (25 mM glucose) and treated with vehicle (DMSO; Cont) or 5 µM NCGC00244536 (NC536) for 16 h. (**A**–**D**) Intracellular pyruvate dehydrogenase (PDH) activity was measured and normalized to total protein concentration determined by the BCA assay (*N* = 4). (**E**–**H**) Extracellular pyruvate levels were quantified and normalized to viable cell number determined in parallel cultures using the WST-8 assay (*N* = 4). (**I**–**L**) Intracellular ATP levels were quantified and normalized to viable cell number determined in parallel cultures using the CellTiter-Glo Cell Viability Assay (*N* = 4). Data are presented as mean ± SEM and expressed as fold change relative to the corresponding vehicle control group (set to 1.0). Statistical significance was determined using an unpaired two-tailed Student’s *t*-test. * *p* < 0.05; ** *p* < 0.01; **** *p* < 0.0001 compared with the corresponding vehicle control group. *N* denotes the number of independent biological replicates from one representative experiment.

To determine whether suppression of PDC expression translated into functional metabolic impairment, we next examined PDH enzymatic activity together with downstream metabolic consequences following pharmacological inhibition of KDM4B (Figure 6). PDH activity was normalized to total protein concentration in accordance with the assay protocol, whereas extracellular pyruvate and intracellular ATP levels were normalized to viable cell number determined in parallel cultures to minimize the influence of treatment-induced differences in cell viability.

Consistent with the reduction in PDC expression, treatment with 5 µM NCGC00244536 significantly decreased PDH enzymatic activity in all three cancer cell lines, whereas no significant change was observed in non-malignant HK-2 cells (Figure 6A–D). Relative PDH activity was reduced by approximately 40–50% in HCT-116, HeLa, and G-361 cells compared with their respective vehicle-treated controls.

To determine whether reduced PDH activity altered pyruvate utilization, we next measured extracellular pyruvate accumulation. NCGC00244536 treatment significantly increased extracellular pyruvate accumulation in HCT-116, HeLa, and G-361 cells (Figure 6F–H), consistent with impaired utilization of pyruvate following suppression of PDH activity. In contrast, extracellular pyruvate levels were modestly but significantly reduced in HK-2 cells following NCGC00244536 treatment (Figure 6E), indicating a distinct metabolic response in non-malignant cells.

Finally, we evaluated the impact of KDM4B inhibition on cellular energy production by measuring intracellular ATP levels. Consistent with reduced PDH activity and increased extracellular pyruvate accumulation, intracellular ATP levels were significantly decreased in all three cancer cell lines following NCGC00244536 treatment (Figure 6J–L). In contrast, ATP levels remained unchanged in HK-2 cells despite drug treatment (Figure 6I).

Overall, these findings demonstrate that pharmacological inhibition of KDM4B impairs PDH activity and disrupts pyruvate metabolism in cancer cells, resulting in reduced ATP levels. In contrast, non-malignant HK-2 cells maintained PDH activity and intracellular ATP levels while exhibiting a distinct pattern of extracellular pyruvate regulation, suggesting that normal cells respond differently to KDM4B inhibition.

### 2.7. Pharmacological Inhibition of KDM4B Selectively Impairs Glucose Uptake in Cancer Cells

**Figure 7:**
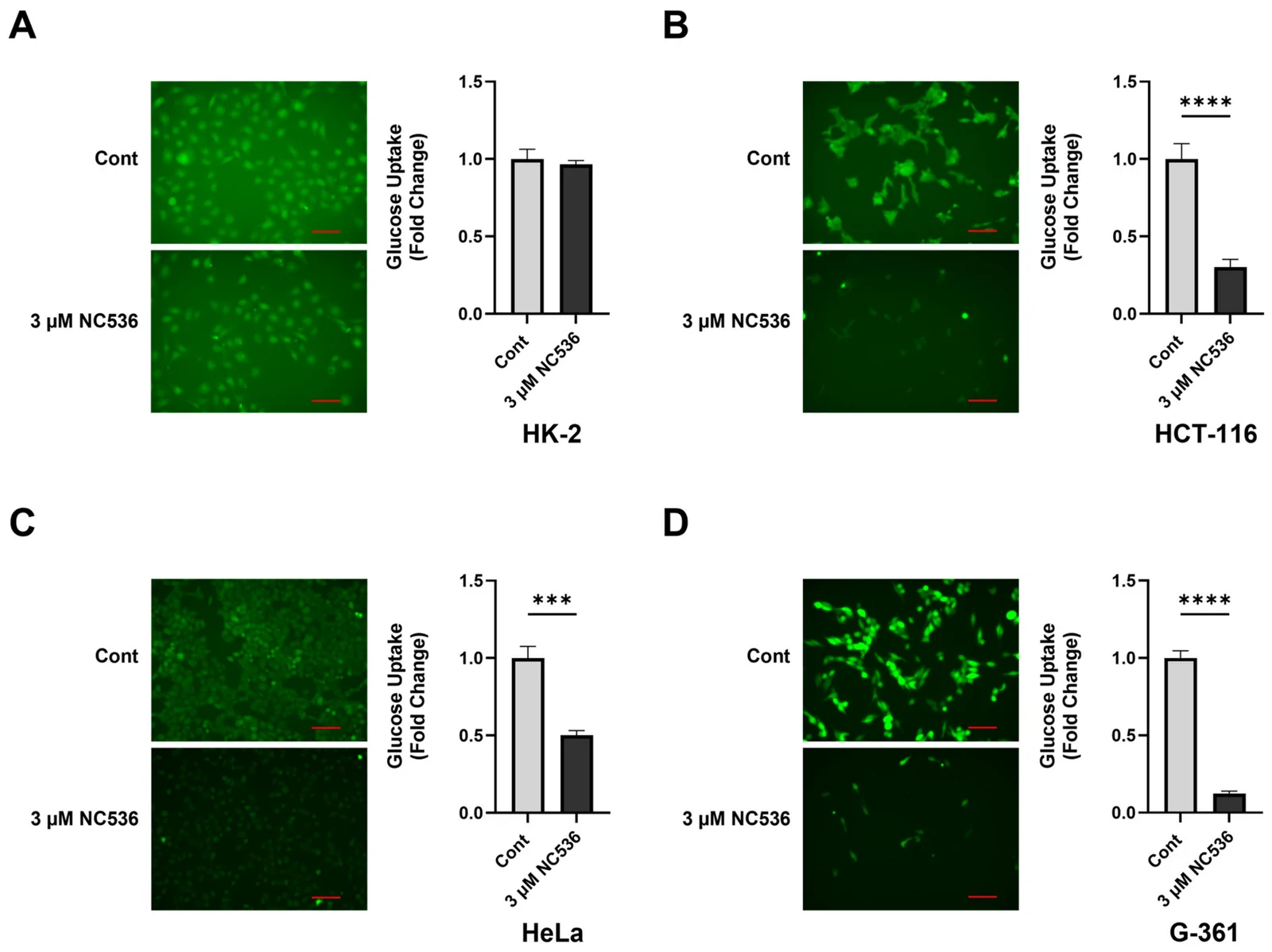
Pharmacological inhibition of KDM4B selectively impairs glucose uptake in cancer cells. Human non-malignant proximal tubule epithelial cells (HK-2) and human cancer cell lines (HCT-116, HeLa, and G-361) were cultured in high-glucose medium (25 mM glucose) and treated with vehicle (DMSO; Cont) or a sub-toxic concentration of 3 µM NCGC00244536 (NC536) for 16 h. (**A**–**D**) Representative fluorescence microscopy images (**left**) and corresponding quantitative analysis (**right**) of cellular glucose uptake based on intracellular green fluorescence intensity (*N* = 7). Scale bar, 100 µm. Data are presented as mean ± SEM and expressed as fold change relative to the corresponding vehicle control group (set to 1.0). Statistical significance was determined using an unpaired two-tailed Student’s *t*-test. *** *p* < 0.001; **** *p* < 0.0001 compared with the corresponding vehicle control group. *N* denotes the number of independently acquired microscopic fields obtained from multiple biological replicates from one representative experiment.

Because KDM4B inhibition impaired mitochondrial glucose metabolism, we next examined whether glucose uptake was also altered, and evaluated the uptake of a fluorescent glucose analogue following treatment with NCGC00244536 (Figure 7). A concentration of 3 µM NCGC00244536 was used for these imaging experiments to preserve cell morphology while allowing for reliable quantification of intracellular fluorescence.

Fluorescence microscopy revealed a marked reduction in glucose uptake in all three cancer cell lines following NCGC00244536 treatment (Figure 7B–D). Quantitative analysis demonstrated that intracellular fluorescence intensity decreased by approximately 70% in HCT-116 cells, 50% in HeLa cells, and 85% in G-361 cells compared with the corresponding vehicle-treated controls. In contrast, glucose uptake in non-malignant HK-2 cells remained unchanged following treatment (Figure 7A).

These findings indicate that pharmacological inhibition of KDM4B selectively impairs glucose uptake in cancer cells while exerting minimal effects on non-malignant HK-2 cells, further supporting the preferential disruption of glucose metabolism in malignant cells.

### 2.8. Pharmacological Inhibition of KDM4B Disrupts Mitochondrial Membrane Potential

**Figure 8:**
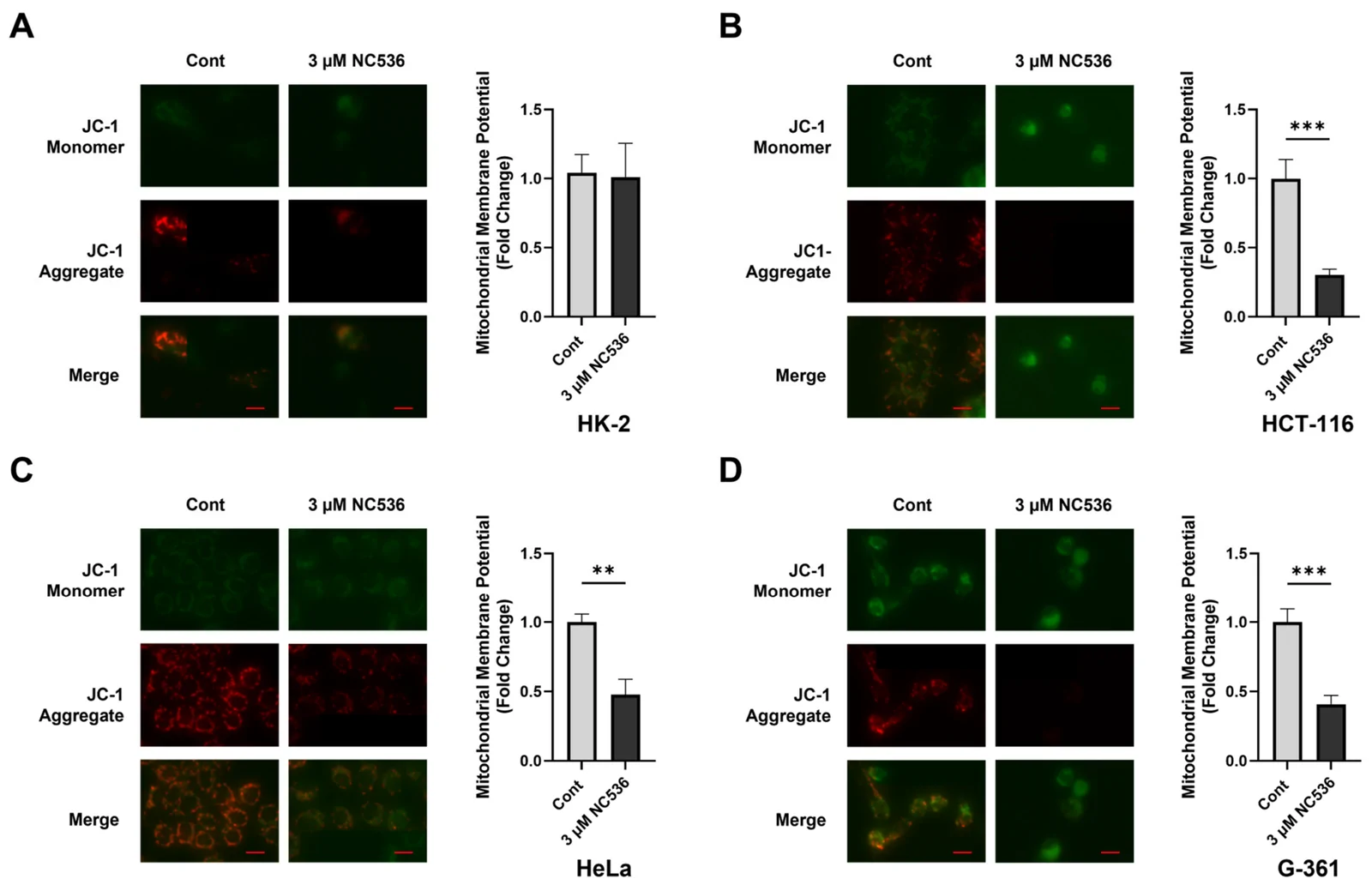
Pharmacological inhibition of KDM4B selectively disrupts mitochondrial membrane potential in cancer cells. Human non-malignant proximal tubule epithelial cells (HK-2) and human cancer cell lines (HCT-116, HeLa, and G-361) were cultured in high-glucose medium (25 mM glucose) and treated with vehicle (DMSO; Cont) or a sub-toxic concentration of 3 µM NCGC00244536 (NC536) for 16 h. (**A**–**D**) Representative fluorescence microscopy images (left) showing JC-1 monomers (green), JC-1 aggregates (red), and merged images, together with the corresponding quantitative analysis (right) of mitochondrial membrane potential expressed as the JC-1 red-to-green fluorescence intensity ratio (*N* = 6). Scale bar, 25 µm. Data are presented as mean ± SEM and expressed as fold change relative to the corresponding vehicle control group (set to 1.0). Statistical significance was determined using an unpaired two-tailed Student’s *t*-test. ** *p* < 0.01; *** *p* < 0.001 compared with the corresponding vehicle control group. *N* denotes the number of independently acquired microscopic fields obtained from multiple biological replicates from one representative experiment.

Given the marked disruption of glucose metabolism and ATP production following KDM4B inhibition, we next evaluated mitochondrial membrane potential (ΔΨm) using the ratiometric fluorescent probe JC-1 (Figure 8). Cells were treated with 3 µM NCGC00244536 for 16 h under high-glucose conditions, and mitochondrial membrane potential was assessed by measuring the ratio of red fluorescent JC-1 aggregates to green fluorescent JC-1 monomers.

Representative fluorescence images demonstrated a marked reduction in mitochondrial membrane potential in all three cancer cell lines following NCGC00244536 treatment (Figure 8B–D). Vehicle-treated cells exhibited predominantly red JC-1 aggregates, indicative of polarized mitochondria, whereas NCGC00244536 treatment resulted in a pronounced increase in green JC-1 monomer fluorescence together with a corresponding reduction in red fluorescence. Quantitative analysis confirmed significant decreases in the red-to-green fluorescence ratio in HCT-116, HeLa, and G-361 cells, indicating loss of mitochondrial membrane potential (Figure 8B–D). In contrast, NCGC00244536 treatment produced no significant changes in JC-1 fluorescence distribution or the red-to-green fluorescence ratio in non-malignant HK-2 cells, indicating preservation of mitochondrial membrane potential under the same experimental conditions (Figure 8A).

Taken together, these findings demonstrate that pharmacological inhibition of KDM4B selectively disrupts mitochondrial membrane potential in cancer cells while sparing non-malignant HK-2 cells. These observations extend the metabolic defects identified in the preceding experiments and further support the conclusion that inhibition of the KDM4B–PDC axis is associated with impaired mitochondrial function in malignant cells.

### 2.9. Pharmacological Inhibition of KDM4B Selectively Suppresses Cancer Cell Viability and Proliferative Capacity

**Figure 9:**
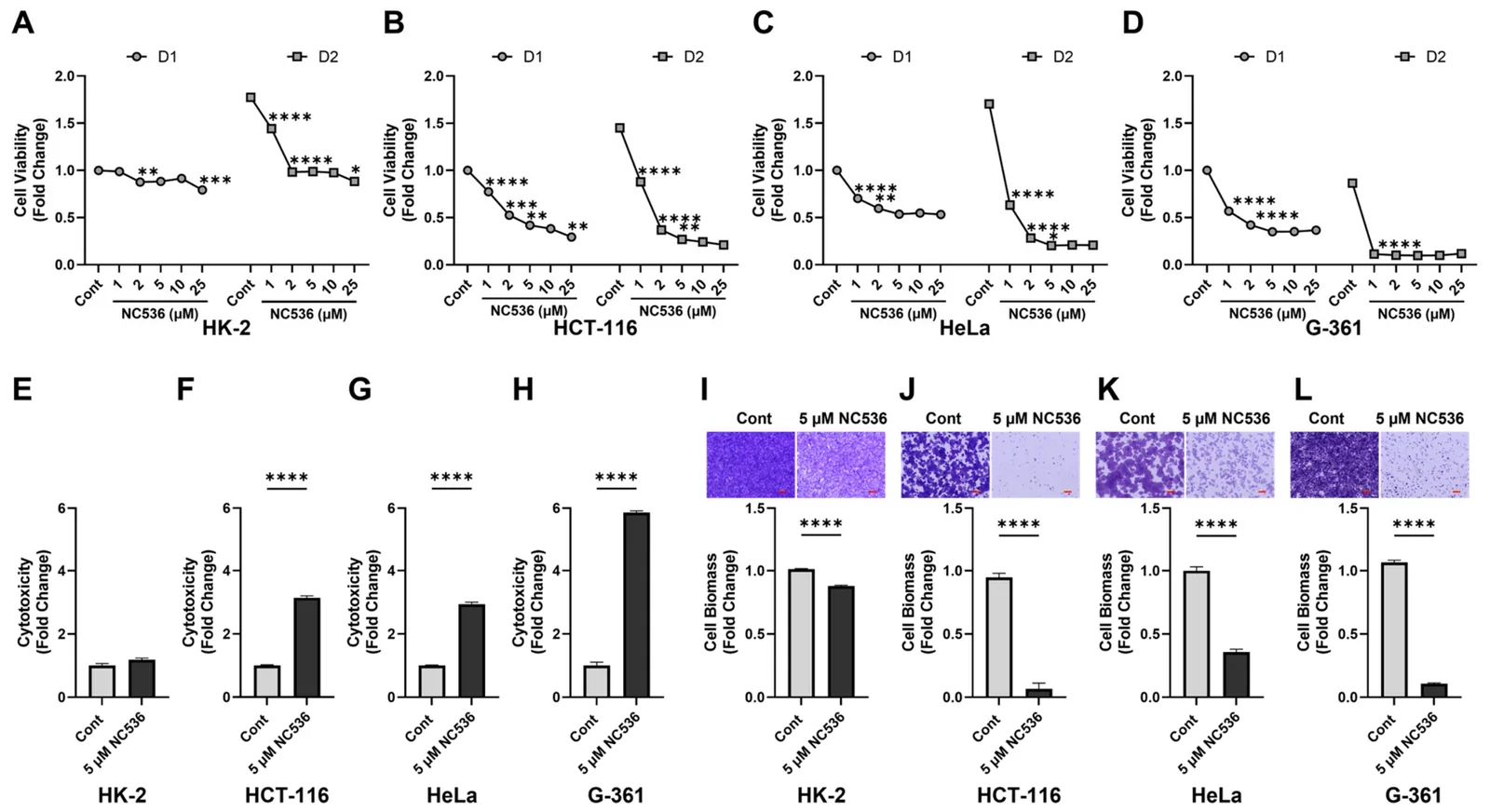
Pharmacological inhibition of KDM4B selectively suppresses cancer cell viability, enhances cytotoxicity, and reduces cell biomass. Human non-malignant proximal tubule epithelial cells (HK-2) and human cancer cell lines (HCT-116, HeLa, and G-361) were cultured in high-glucose medium (25 mM glucose) and treated with vehicle (DMSO; Cont) or NCGC00244536 (NC536). (**A**–**D**) Cell viability was assessed using the WST-8 assay at 24 h (D1) and 48 h (D2) following treatment with increasing concentrations of NCGC00244536 (1, 2, 5, 10, and 25 µM) (*N* = 4). (**E**–**H**) Cellular cytotoxicity was evaluated by measuring lactate dehydrogenase (LDH) release into the culture supernatant following 16 h of treatment with vehicle (DMSO; Cont) or 5 µM NCGC00244536 (*N* = 4). (**I**–**L**) Cells were treated with vehicle (DMSO; Cont) or 5 µM NCGC00244536 for 16 h, after which the treatment medium was replaced with fresh complete culture medium and cells were cultured until the control group reached 80–90% confluency. Representative crystal violet-stained cultures and the corresponding quantitative analysis of cell biomass are shown (*N* = 7). Scale bar, 300 µm. Data are presented as mean ± SEM and expressed as fold change relative to the corresponding vehicle control group (set to 1.0). Statistical significance was determined using two-way ANOVA followed by Tukey’s multiple-comparisons test for panels (**A**–**D**), and an unpaired two-tailed Student’s *t*-test for panels (**E**–**L**). * *p* < 0.05; ** *p* < 0.01; *** *p* < 0.001; **** *p* < 0.0001 compared with the corresponding vehicle control group. *N* denotes the number of independent biological replicates (**A**–**H**) or independently acquired microscopic fields obtained from multiple biological replicates (**I**–**L**) from one representative experiment.

To determine whether pharmacological inhibition of KDM4B phenocopies genetic depletion, non-malignant HK-2 cells and three cancer cell lines (HCT-116, HeLa, and G-361) were treated with NCGC00244536, and cell viability was assessed over a range of concentrations (Figure 9A–D).

NCGC00244536 reduced cell viability in a dose- and time-dependent manner in all three cancer cell lines, whereas HK-2 cells exhibited substantially lower sensitivity to the treatment. At 48 h (D2), treatment with 2–5 µM NCGC00244536 reduced the viability of HCT-116, HeLa, and G-361 cells to below 30% of the corresponding vehicle-treated controls, with further reductions observed at higher concentrations (Figure 9B–D). In contrast, HK-2 cells maintained near-baseline viability at 1–2 µM NCGC00244536, and approximately 50% viability was retained even at the highest concentration tested (25 µM) (Figure 9A).

To determine whether the reduction in cell viability was accompanied by increased cytotoxicity, lactate dehydrogenase (LDH) release was measured following 16 h of treatment with 5 µM NCGC00244536. LDH release increased significantly in all three cancer cell lines, whereas no significant increase was detected in HK-2 cells under the same treatment conditions (Figure 9E–H).

We next examined whether transient pharmacological inhibition of KDM4B produced sustained effects on proliferative capacity. Following a 16 h exposure to NCGC00244536, the treatment medium was replaced with fresh complete medium, and cells were cultured until the vehicle-treated controls reached 80–90% confluency. Crystal violet staining revealed only a modest reduction in cell biomass in HK-2 cells (Figure 9I), whereas HCT-116, HeLa, and G-361 cells exhibited a marked reduction in cell biomass, with approximately 90% suppression relative to their respective vehicle controls (Figure 9J–L).

Overall, these findings demonstrate that pharmacological inhibition of KDM4B phenocopies the effects of genetic depletion by selectively reducing cell viability, increasing cytotoxicity, and suppressing proliferative recovery in cancer cells while exerting comparatively limited effects on non-malignant HK-2 cells.

## 3. Discussion

### 3.1. KDM4B Is Associated with the Regulation of the Pyruvate Dehydrogenase Complex in Cancer Cells

The present study identifies KDM4B as a previously unrecognized epigenetic regulator associated with the pyruvate dehydrogenase complex (PDC) in multiple cancer cell models. Although KDM4B has been extensively implicated in tumor progression, metabolic adaptation, and transcriptional regulation [10,12,17,18], its role in coordinating expression of the core PDC components has not previously been established. Here, both genetic depletion and pharmacological inhibition of KDM4B consistently suppressed the expression of PDHA1, DLD, and DLAT, demonstrating that KDM4B contributes to maintenance of the PDC at the transcriptional level. These transcriptional changes were accompanied by reduced PDH protein abundance, diminished PDH enzymatic activity, mitochondrial depolarization, decreased ATP production, and selective inhibition of cancer cell proliferation, establishing the KDM4B–PDC axis as a previously unrecognized mechanism linking epigenetic regulation to mitochondrial glucose metabolism.

Previous studies have primarily focused on the ability of KDM4B to regulate the PDK–PDH signaling axis, thereby modulating PDH phosphorylation and enzymatic activity. In colorectal cancer, KDM4B has been reported to promote malignant progression through transcriptional regulation of oncogenic pathways, supporting its broader role as an important epigenetic regulator of tumor biology [14]. Likewise, Li et al. demonstrated that KDM4B is required to sustain glycolytic metabolism in colorectal cancer cells [14], whereas Wu et al. reported that KDM4B depletion enhances PDH activity through downregulation of PDKs in castration-resistant prostate cancer [15]. Our findings extend these observations by suggesting that KDM4B contributes to mitochondrial glucose metabolism at multiple levels. In addition to its reported association with the PDK/PDP-PDH regulatory axis, our findings indicate that KDM4B contributes to maintenance of the structural components of the PDC itself. Consequently, KDM4B inhibition reduces both the abundance of the enzyme complex and its catalytic activity, providing an explanation for the marked reductions in ATP production observed in the present study despite potential compensatory mechanisms affecting PDH phosphorylation.

The glucose-response experiments further support this model. Glucose availability induced KDM4B expression in the cancer cell models examined, accompanied by increased expression of PDC genes and changes in the total PDH and phosphorylated PDH (Ser293) signals. Conversely, pharmacological inhibition of KDM4B suppressed PDC gene expression, was associated with reduced PDH protein abundance and reduced PDH enzymatic activity, together with impaired glucose uptake, mitochondrial depolarization, decreased ATP production, and increased apoptotic signaling. Together, these findings suggest that KDM4B functions as an important regulator of mitochondrial glucose utilization required for maintaining the metabolic phenotype of cancer cells.

The RNA-seq analysis provides additional mechanistic context for these observations by demonstrating coordinated transcriptional remodeling across multiple metabolic pathways following KDM4B inhibition. Rather than affecting a single metabolic enzyme, inhibition of KDM4B simultaneously reduced expression of the core PDC components (*PDHA1*, *DLAT*, and *DLD*), increased *PDK1*, reduced *PDP1*, and was accompanied by broad suppression of genes involved in the tricarboxylic acid cycle and mitochondrial electron transport. In contrast, glycolytic genes, including *SLC2A1*, *GPI*, *GAPDH*, *ENO1*, *ALDOA*, and *LDHA*, were generally upregulated. Reactome pathway analysis showed the same overall pattern, with positive enrichment trends for glycolysis and glucose metabolism and negative enrichment trends for respiratory electron transport, ATP formation by chemiosmotic coupling, and the TCA cycle. Although these pathway enrichments did not reach statistical significance because the RNA-seq experiment lacked biological replicates, the transcriptomic changes were consistent with the independent biochemical findings, including reduced PDH activity, diminished ATP production, impaired glucose uptake, and mitochondrial depolarization. Collectively, these data are consistent with a model in which KDM4B maintains mitochondrial glucose oxidation through coordinated regulation of both the PDC and downstream oxidative metabolism.

An interesting observation was that pharmacological inhibition increased KDM4B transcript and protein levels despite suppressing KDM4B-dependent metabolic functions. RNA sequencing demonstrated increased KDM4B transcript abundance, which was accompanied by increased KDM4B protein signals in both whole-cell and nuclear lysates, indicating that this adaptive response occurs, at least in part, at the transcriptional level. Nevertheless, repressive H3K9 trimethylation accumulated concurrently, indicating that increased KDM4B expression did not restore demethylase activity. These findings indicate that increased KDM4B abundance does not reflect restoration of enzymatic function. One possible explanation is the activation of a compensatory feedback mechanism, whereby cells attempt to overcome loss of KDM4B catalytic activity by increasing KDM4B expression. Similar adaptive responses have been reported for epigenetic regulators following pharmacological inhibition, where increased protein abundance does not necessarily restore enzymatic activity because inhibitor binding remains intact [19]. Accordingly, restoration of KDM4B expression in our study was insufficient to reverse H3K9 trimethylation or rescue PDC expression, indicating that catalytic inhibition, rather than protein abundance, determines the downstream metabolic phenotype.

Our findings further suggest that suppression of PDC expression is associated with restoration of repressive histone methylation. Pharmacological inhibition of KDM4B increased nuclear H3K9 trimethylation while simultaneously reducing PDC expression, consistent with the established role of KDM4B as an H3K9 demethylase that promotes transcriptional activation [20,21]. Although the present study does not directly demonstrate promoter-specific regulation, the coordinated reduction in multiple PDC genes together with increased H3K9 trimethylation is consistent with a model in which KDM4B contributes to maintenance of PDC transcription through epigenetic regulation. Future chromatin immunoprecipitation (ChIP) studies will be required to determine whether PDHA1, DLD, and DLAT are direct transcriptional targets of KDM4B.

### 3.2. Functional Disruption of the KDM4B–PDC Axis Selectively Impairs Cancer Cell Metabolism

The molecular changes observed following KDM4B inhibition were accompanied by marked functional alterations in cellular metabolism. Suppression of PDC expression resulted in reduced PDH enzymatic activity, increased extracellular pyruvate accumulation, and decreased intracellular ATP production, indicating impaired mitochondrial pyruvate utilization [3]. Pyruvate accumulation is a hallmark of a metabolic block at the PDC, which forces cells to divert carbon flux toward lactate or exit the cell [3]. Together with the observed reduction in glucose uptake and loss of mitochondrial membrane potential, these findings demonstrate that inhibition of KDM4B produces coordinated metabolic dysfunction extending from nutrient utilization to mitochondrial integrity [6,9,22,23].

Mitochondrial dysfunction is a well-recognized trigger of intrinsic apoptosis [24,25], and consistent with this concept, disruption of the KDM4B–PDC axis was accompanied by activation of cleaved caspase-3 together with reduced cell viability, increased LDH release, and marked suppression of long-term proliferative capacity. Rather than representing isolated cellular events, these observations suggest a progressive sequence in which epigenetic suppression of the PDC is followed by metabolic dysfunction, mitochondrial impairment, and ultimately apoptotic cell death.

In this regard, Shi et al. (2021) identified a [Ca^2+^]m-PDP1-PDH retrograde signaling axis in colorectal cancer where the downregulation of active PDH drives aerobic glycolysis and enhances DNA damage repair as a survival adaptation [16]. Our findings suggest that KDM4B functions as an important coordinator of this metabolic–epigenetic crosstalk. Our data further suggest that inhibition of KDM4B impairs two complementary metabolic processes. In addition to suppressing mitochondrial pyruvate utilization through downregulation of the PDC, KDM4B inhibition markedly reduced glucose uptake, indicating that both mitochondrial glucose oxidation and cellular glucose utilization are compromised simultaneously. Consequently, KDM4B inhibition appears to compromise the metabolic flexibility of cancer cells by simultaneously impairing mitochondrial glucose oxidation and glucose uptake. This combined metabolic disruption may explain the pronounced reduction in ATP production and the subsequent activation of apoptotic signaling observed in the present study.

### 3.3. Differential Metabolic Responses Reveal a Potential Therapeutic Window

One of the most notable observations of this study was the striking difference between malignant and non-malignant cells following KDM4B inhibition. Whereas cancer cells consistently exhibited suppression of PDC expression, metabolic dysfunction, mitochondrial depolarization, and apoptotic activation, these changes were largely absent in HK-2 cells.

In contrast, KDM4B inhibition reduced extracellular pyruvate accumulation while maintaining intracellular ATP levels in HK-2 cells. Although extracellular pyruvate accumulation in cancer cells is consistent with a “bottleneck” in mitochondrial pyruvate oxidation, the distinct response in HK-2 cells suggests they possess greater metabolic flexibility. This capability may enable non-malignant cells to compensate through alternative substrates, such as fatty acids or amino acids, when glucose-derived mitochondrial metabolism is perturbed [3,26]. These observations are consistent with the concept that non-malignant cells possess greater metabolic plasticity, allowing them to compensate for impaired glucose oxidation through alternative energy substrates [3,27].

Consequently, future isotope-tracing studies and metabolic flux analyses will be valuable for defining how inhibition of the KDM4B–PDC axis alters carbon utilization through the tricarboxylic acid cycle and associated biosynthetic pathways [3,27]. The differential sensitivity observed across colorectal, cervical, and melanoma cell models suggests that the KDM4B–PDC axis may represent a conserved metabolic dependency in multiple cancer cells. Although further validation in animal models will be necessary, the preservation of metabolic homeostasis in HK-2 cells supports the possibility that KDM4B may represent a therapeutically relevant metabolic vulnerability, although tissue-matched and in vivo studies are required to assess selectivity and safety [27].

### 3.4. Study Limitations and Future Perspectives

Several limitations should be considered when interpreting the present findings. First, the precise epigenetic mechanism linking KDM4B to PDC gene expression remains to be established. Although KDM4B inhibition increased H3K9me3 concomitantly with suppression of PDHA1, DLD, and DLAT, locus-specific chromatin occupancy was not examined. ChIP-based studies will therefore be required to determine whether these genes are direct targets of KDM4B-dependent chromatin regulation. In addition, the RNA-seq analysis was exploratory because biological replicates were not available; its pathway-level findings were therefore interpreted together with independent biochemical and functional measurements.

Second, the present data support, but do not fully establish, a causal link between PDC suppression, mitochondrial dysfunction, and cell survival. KDM4B inhibition reduced PDC expression and PDH activity and was accompanied by ATP depletion and mitochondrial depolarization, providing functional evidence of impaired mitochondrial metabolic homeostasis. However, direct measurements of respiratory capacity and functional rescue of PDC activity were not performed. Independent KDM4B perturbation and PDC rescue studies will therefore be important to define the causal contribution of the KDM4B–PDC axis to mitochondrial and survival phenotypes.

Third, the study was performed using two-dimensional cancer cell models and a single non-malignant comparator. Although the consistent metabolic phenotype across colorectal, cervical, and melanoma cell lines supports the broader relevance of KDM4B–PDC regulation, the use of non-tissue-matched HK-2 cells does not establish cancer selectivity. Moreover, three-dimensional and in vivo models will be required to determine how the KDM4B–PDC axis operates within the tumor microenvironment and to evaluate its therapeutic relevance and systemic safety.

## 4. Materials and Methods

### 4.1. Cell Lines, Cell Culture, and Reagents

Human colorectal carcinoma (HCT-116), cervical adenocarcinoma (HeLa), malignant melanoma (G-361), and immortalized human proximal tubule epithelial (HK-2) cell lines were obtained from the American Type Culture Collection (ATCC, Manassas, VA, USA). HCT-116, HeLa, and G-361 cells were used as representative cancer cell models, whereas HK-2 cells served as a non-malignant epithelial control.

HCT-116, HeLa, and G-361 cells were maintained in Dulbecco’s Modified Eagle Medium (DMEM) containing 25 mM glucose (D6429, Sigma-Aldrich, St. Louis, MO, USA). HK-2 cells were cultured in DMEM/F12 medium containing 17.5 mM glucose (11320033, Thermo Fisher Scientific, Waltham, MA, USA). All media were supplemented with 10% fetal bovine serum (FBS; S1810-500, Biowest, Nuaillé, France) and 1% Penicillin–Streptomycin–Glutamine solution (06168-34, Nacalai Tesque, Kyoto, Japan). Cells were maintained at 37 °C in a humidified incubator with 5% CO_2_ and passaged at approximately 50–60% confluence.

Unless otherwise indicated, the day after seeding, cells were incubated in glucose-free DMEM (11966025, Thermo Fisher Scientific, Waltham, MA, USA) supplemented with 0.5% FBS and 1% Penicillin–Streptomycin–Glutamine solution for 6 h to synchronize metabolic conditions. The medium was then replaced with glucose-free or 25 mM glucose-containing DMEM supplemented with 0.5% FBS and 1% Penicillin–Streptomycin–Glutamine solution for the indicated treatment periods [27]. For glucose dose–response experiments, low-glucose DMEM (5 mM glucose; D6046, Sigma-Aldrich, St. Louis, MO, USA) was used as the basal medium, and sterile-filtered D-glucose (049-31165, Fujifilm Wako Pure Chemical, Osaka, Japan) was added to achieve the desired final glucose concentrations.

For pharmacological experiments, cells were treated with the selective KDM4B inhibitor NCGC00244536 (18478, Cayman Chemical, Ann Arbor, MI, USA). Vehicle control groups received an equivalent volume of dimethyl sulfoxide (DMSO; 13408-64, Nacalai Tesque, Kyoto, Japan). Unless otherwise specified, all experimental measurements were performed following 16 h of the final intervention.

### 4.2. RNA Sequencing and Transcriptomic Analysis

HCT-116 cells were cultured under three experimental conditions: glucose-free medium (0 mM glucose), high-glucose medium (25 mM glucose), and high-glucose medium supplemented with the selective KDM4B inhibitor NCGC00244536 (5 μM). After treatment, total RNA was isolated and submitted to Takara Bio Inc. (Kusatsu, Japan) for RNA sequencing. Library preparation, sequencing, read alignment, and transcript quantification were performed by the service provider using their standard analysis pipeline. Gene expression was reported as both transcripts per million (TPM) and normalized read counts.

The processed gene expression table provided by Takara Bio was imported into RStudio (version 2026.01.0 Build 392) for downstream analyses. Genes with TPM values < 1 in all three experimental conditions were excluded to reduce background noise. Gene expression changes between glucose-free and high-glucose conditions, and between high-glucose and NCGC00244536-treated cells, were estimated using log_2_ fold changes calculated from normalized read counts after adding a pseudocount of one.

Because RNA sequencing was performed without biological replicates, statistical differential expression analysis (e.g., DESeq2 or edgeR) was not performed. Instead, the transcriptomic dataset was used as an exploratory analysis to identify coordinated changes in metabolic pathways, and all transcriptomic findings were interpreted together with independent biochemical and functional experiments.

To examine pathways relevant to glucose metabolism, a curated list of genes involved in glycolysis, glucose transport, pyruvate metabolism, the pyruvate dehydrogenase complex, the tricarboxylic acid (TCA) cycle, and mitochondrial electron transport was assembled based on the published literature and Reactome pathway annotations. Heatmaps were generated using the pheatmap R package after row-wise Z-score normalization of TPM values.

To assess pathway-level alterations without imposing arbitrary fold-change thresholds, Gene Set Enrichment Analysis (GSEA) was performed using the clusterProfiler R package with Reactome pathway annotations obtained through the ReactomePA package. Genes were ranked according to the log_2_ fold change between high-glucose and NCGC00244536-treated cells. Pathways related to glucose metabolism, glycolysis, the TCA cycle, respiratory electron transport, and oxidative phosphorylation were evaluated. Given the absence of biological replicates, GSEA results were interpreted qualitatively, with emphasis placed on the direction and consistency of pathway enrichment rather than statistical significance.

All analyses were performed using RStudio (version 2026.01.0 Build 392), and figures were generated using the pheatmap (version 1.0.13), ggplot2 (version 4.0.3), and enrichplot (version 1.26.6) packages.

### 4.3. Gene Silencing by siRNA Transfection

Gene silencing was performed as previously described [28]. Briefly, cells were seeded in 24-well plates, and the following day the culture medium was replaced with 500 µL of fresh complete medium per well. Cells were transfected with 50 nM of either *KDM4B*-specific siRNA (MSS208418, Thermo Fisher Scientific, Waltham, MA, USA) or a non-targeting negative control siRNA (Medium GC Duplex #2, 465372, Thermo Fisher Scientific, Waltham, MA, USA) using 1.5 µL of Lipofectamine RNAiMAX Transfection Reagent (13778, Thermo Fisher Scientific, Waltham, MA, USA) diluted in 100 µL of Opti-MEM I Reduced Serum Medium (31985062, Thermo Fisher Scientific, Waltham, MA, USA), according to the manufacturer’s instructions. The culture medium was replaced 24 h after transfection, and downstream analyses were performed 4–5 days post-transfection.

### 4.4. Total RNA Extraction, cDNA Synthesis, and Reverse Transcription Quantitative PCR (RT-qPCR)

Following the appropriate interventions, total RNA was extracted and reverse transcription quantitative PCR (RT-qPCR) was performed as previously described [11,28]. Total RNA was isolated using TRIzol reagent (15596018, Thermo Fisher Scientific, Waltham, MA, USA) according to the manufacturer’s instructions. First-strand cDNA was synthesized using the PrimeScript RT Reagent Kit (RR037A, Takara Bio, Kusatsu, Japan). Quantitative PCR was performed using PowerTrack SYBR Green Master Mix (A46109, Thermo Fisher Scientific Baltics UAB, Vilnius, Lithuania) on a 7500 Fast Real-Time PCR System (Thermo Fisher Scientific, Waltham, MA, USA). Primer sequences are listed in Supplementary Table S6.

Gene expression was quantified using the standard curve method based on serially diluted cDNA standards. Target gene expression was normalized to the geometric mean of the reference genes TATA-box binding protein (TBP) and 18S ribosomal RNA (18S rRNA) to improve normalization accuracy. Normalized expression values were calculated relative to the corresponding control group and are presented as fold change.

### 4.5. SDS-PAGE and Western Blotting

Whole-cell and nuclear protein lysates were prepared using RIPA buffer (89900, Thermo Fisher Scientific, Waltham, MA, USA) and NE-PER Nuclear and Cytoplasmic Extraction Reagents (78833, Thermo Fisher Scientific, Waltham, MA, USA), respectively. Both extraction buffers were supplemented with Halt Protease Inhibitor Cocktail (78430, Thermo Fisher Scientific, Waltham, MA, USA) according to the manufacturer’s instructions. Protein samples were separated on 10% polyacrylamide gels prepared using the TGX FastCast Acrylamide Kit (1610171, Bio-Rad Laboratories, Hercules, CA, USA) and transferred onto nitrocellulose membranes (Immobilon-P, IPVH00005, Merck Millipore, Cork, Ireland).

The following primary antibodies were used: anti-KDM4B/JMJD2B (DF13288, Affinity Biosciences, Cincinnati, OH, USA); anti-phospho-pyruvate dehydrogenase E1α (Ser293) (37115), anti-pyruvate dehydrogenase (3205), anti-DLAT (12362), anti-cleaved caspase-3 (9664), anti-caspase-3 (14220), and anti-β-tubulin (2128) (all from Cell Signaling Technology, Danvers, MA, USA); anti-H3K9me3 (A8A1) and anti-Lamin A/C (D9G3) (both from Selleck Chemicals, Yokohama, Japan).

Protein bands were visualized using an Odyssey Fc Imaging System (LI-COR Biosciences, Lincoln, NE, USA) with chemiluminescent detection and analyzed using Image Studio Lite software (version 5.2, LI-COR Biosciences, Lincoln, NE, USA) as previously described [11,29].

### 4.6. WST-8 Cell Viability Assay

Cell viability was assessed using a WST-8 colorimetric assay (Cell Counting Kit-8; 347-07621, Dojindo Laboratories, Mashiki, Japan). Cells were seeded in 96-well plates and treated as indicated. For NCGC00244536-treated groups, viability was measured 24 or 48 h after treatment. For siRNA-transfected groups, viability was assessed 5 days post-transfection. WST-8 reagent was added directly to the culture medium according to the manufacturer’s instructions, followed by incubation at 37 °C for 30 min. Absorbance was measured at 450 nm using a Spark multimode microplate reader (Tecan Austria GmbH, Grödig, Austria).

### 4.7. CellTiter-Glo Luminescent Cell Viability Assay

Cell viability was independently evaluated using the CellTiter-Glo Luminescent Cell Viability Assay (G9242, Promega, Madison, WI, USA) according to the manufacturer’s instructions. The reagent was added directly to the culture wells to induce cell lysis, and luminescence was measured after 5 min of incubation at room temperature using a Spark multimode microplate reader (Tecan Austria GmbH, Grödig, Austria). This assay was used both to assess cell viability and, where indicated, to determine viable cell number for normalization of intracellular ATP measurements.

### 4.8. Crystal Violet Staining

Cell biomass was assessed by crystal violet staining (038-04862, Fujifilm Wako Pure Chemical, Osaka, Japan). Cells were seeded in 24-well plates and treated as indicated. Following treatment, cells were washed twice with phosphate-buffered saline (PBS) and fixed/stained with 0.5% crystal violet prepared in methanol at −20 °C for 30 min. Excess stain was removed by washing with distilled water, and plates were air-dried overnight. Bright-field images were acquired using a BZ-X710 microscope (Keyence, Osaka, Japan). Cell biomass was quantified as the integrated staining density using BZ-X Analyzer software (version 1.4.1.1, Keyence, Osaka, Japan).

### 4.9. Lactate Dehydrogenase (LDH) Cytotoxicity Assay

Cellular cytotoxicity was evaluated by measuring lactate dehydrogenase (LDH) release into the culture medium using the CyQUANT LDH Cytotoxicity Assay Kit (C20300, Thermo Fisher Scientific, Waltham, MA, USA) according to the manufacturer’s instructions. Absorbance was measured at 490 nm with background correction at 680 nm using a Spark multimode microplate reader (Tecan Austria GmbH, Grödig, Austria). LDH activity was calculated by subtracting the 680-nm reference absorbance from the corresponding 490-nm absorbance.

### 4.10. Intracellular ATP Quantification

Intracellular ATP levels were quantified using an ATP Assay Kit-Luminescence (340-09791, Dojindo Laboratories, Mashiki, Japan). Cells were seeded in 96-well plates and treated as indicated. Following treatment, intracellular ATP was measured from cell lysates according to the manufacturer’s instructions, and luminescence was recorded using a Spark multimode microplate reader (Tecan Austria GmbH, Grödig, Austria). To account for treatment-induced differences in cell number, intracellular ATP values were normalized to viable cell number determined in parallel cultures using the CellTiter-Glo Luminescent Cell Viability Assay. Data are expressed as fold change relative to the corresponding control group.

### 4.11. Pyruvate Dehydrogenase (PDH) Activity Assay

Intracellular PDH activity was measured using a Pyruvate Dehydrogenase Activity Assay Kit (MA-PDH, RayBiotech, Peachtree Corners, GA, USA). Cells were cultured in 12-well plates and treated as indicated. Whole-cell lysates were prepared using RIPA buffer supplemented with protease inhibitors. PDH activity was determined by monitoring the MTT-coupled enzymatic reaction according to the manufacturer’s instructions. Total protein concentration was measured using the Pierce BCA Protein Assay Kit (23223 and 23224, Thermo Fisher Scientific, Waltham, MA, USA), and PDH activity was normalized to total protein content as recommended by the assay manufacturer.

### 4.12. Extracellular Pyruvate Quantification

Extracellular pyruvate was quantified using a Pyruvate Assay Kit (700470, Cayman Chemical, Ann Arbor, MI, USA). Cells were seeded in 96-well plates and treated as indicated using pyruvate-free DMEM (D5796, Sigma-Aldrich, St. Louis, MO, USA). Following treatment, culture supernatants were collected and pyruvate concentrations were determined fluorometrically according to the manufacturer’s instructions using a Spark multimode microplate reader (Tecan Austria GmbH, Grödig, Austria). To account for treatment-induced differences in cell number, extracellular pyruvate values were normalized to viable cell number determined in parallel cultures using the WST-8 Cell Viability Assay. Data are expressed as fold change relative to the corresponding control group.

### 4.13. Glucose Uptake Assay

Cellular glucose uptake was evaluated using a fluorescent glucose uptake assay kit (UP02, Dojindo Laboratories, Mashiki, Japan). Cells were cultured in 96-well plates and treated as indicated. Following treatment, cells were incubated with the fluorescent glucose analog according to the manufacturer’s instructions. After removal of excess probe, fluorescence images were acquired using a BZ-X710 fluorescence microscope (Keyence, Osaka, Japan). Intracellular fluorescence intensity was quantified using ImageJ software (version 1.54t, National Institutes of Health, Bethesda, MD, USA).

### 4.14. JC-1 Mitochondrial Membrane Potential Assay

Mitochondrial membrane potential (ΔΨm) was evaluated using the JC-1 MitoMP Detection Kit (349-09401, Dojindo Laboratories, Mashiki, Japan). Cells were cultured in 96-well plates and treated as indicated. Following treatment, cells were incubated with 4 μM JC-1 dye in DMEM for 30 min according to the manufacturer’s instructions. Fluorescence images were acquired using a BZ-X710 fluorescence microscope (Keyence, Osaka, Japan). Red fluorescence (JC-1 aggregates) and green fluorescence (JC-1 monomers) were quantified, and mitochondrial membrane potential was expressed as the ratio of red-to-green fluorescence intensity.

### 4.15. Data Normalization and Statistical Analysis

To facilitate comparison across different analytical platforms, raw absorbance, luminescence, fluorescence, and quantitative PCR data were normalized as described for each assay and expressed as fold change relative to the corresponding control group (set to 1.0). For metabolic assays, intracellular ATP and extracellular pyruvate measurements were normalized to viable cell number determined in parallel cultures, whereas PDH activity was normalized to total protein concentration in accordance with the manufacturer’s protocol.

Data are presented as mean ± standard error of the mean (SEM). Statistical analyses were performed using GraphPad Prism version 9.0.0 (GraphPad Software, San Diego, CA, USA). Comparisons between two groups were performed using an unpaired two-tailed Student’s *t*-test. Comparisons among multiple groups were analyzed by one-way or two-way analysis of variance (ANOVA), followed by Tukey’s multiple-comparisons test where appropriate. The statistical test used for each experiment is specified in the corresponding figure legend. A *p* value < 0.05 was considered statistically significant. Unless otherwise indicated, *N* represents independent biological replicates, and experiments were repeated at least twice using independently cultured cells to confirm reproducibility. For imaging-based experiments, *N* indicates independently acquired microscopic fields from at least three biological replicates, which were analyzed quantitatively as described in the corresponding figure legends.

RNA sequencing was performed as a single exploratory experiment without biological replicates. Consequently, no statistical comparisons of individual genes were performed, and transcriptomic analyses were used to generate mechanistic insights that were validated by independent molecular, biochemical, and functional experiments.

## 5. Conclusions

In summary, the present study identifies KDM4B as a previously unrecognized epigenetic regulator associated with mitochondrial glucose metabolism in cancer cells. We demonstrate that KDM4B contributes to maintaining expression of the pyruvate dehydrogenase complex, thereby supporting PDH activity, mitochondrial function, and cellular energy production. Pharmacological inhibition of KDM4B induced coordinated metabolic reprogramming characterized by suppression of the PDC, reduced mitochondrial oxidative metabolism, impaired glucose uptake, loss of mitochondrial membrane potential, and activation of apoptotic signaling. Importantly, these metabolic defects occurred preferentially in cancer cells, whereas non-malignant HK-2 cells exhibited comparatively limited responses. Collectively, these findings identify the KDM4B–PDC axis as a previously unrecognized metabolic vulnerability in cancer cells and provide a rationale for the further investigation of KDM4B-targeted therapies, either alone or in combination with existing anticancer strategies.

## Data Availability

The data generated and analyzed during the course of current study are available from the corresponding author upon reasonable request.

## Supplementary Materials

The following supporting information can be downloaded at: https://www.mdpi.com/article/10.3390/epigenomes10030059/s1, Table S1: Differential expression of genes involved in pyruvate dehydrogenase regulation and the PDH complex; Table S2: Differential expression of glycolysis-related genes; Table S3: Differential expression of tricarboxylic acid (TCA) cycle genes; Table S4: Differential expression of mitochondrial electron transport chain genes; Table S5: Reactome gene set enrichment analysis of NCGC-treated HCT116 cells; Table S6: List of primers.

## Abbreviations

The following abbreviations are used in this manuscript:

KDM4B	Lysine (K)-specific demethylase 4B
NC536	NCGC00244536
PDC	Pyruvate dehydrogenase complex
TCA	Tricarboxylic acid cycle
PDHA1	Pyruvate dehydrogenase (E1)
DLAT	Dihydrolipoamide acetyltransferase (E2)
DLD	Dihydrolipoamide dehydrogenase (E3)
PDK	Pyruvate dehydrogenase kinases
PDP	Pyruvate dehydrogenase phosphatases
H3K9me3	Histone H3 lysine 9 trimethylation
GLUT1	Glucose transporter 1
OXPHOS	Oxidative Phosphorylation
PDH	PDC subunit PDH E1α
GSEA	Gene Set Enrichment Analysis
RNA-seq	RNA sequencing
